# T1AM Attenuates the Hypoxia/Reoxygenation-Induced Necroptosis of H9C2 Cardiomyocytes via RIPK1/RIPK3 Pathway

**DOI:** 10.1155/2022/4833791

**Published:** 2022-02-28

**Authors:** Bo Wei, Hanbing Zhao, Bailong Hu, Lujun Dai, Guoning Zhang, Lili Mo, Niwen Huang, Changchao Zou, Bei Zhang, Haiyan Zhou, Wei Li, Xingde Liu

**Affiliations:** ^1^Department of Basic Medicine, Guizhou Medical University, Guiyang, Guizhou Province 550004, China; ^2^Department of Cardiovascular Medicine, The Affiliated Hospital of Guizhou Medical University, Guiyang, Guizhou Province 550004, China; ^3^Department of Electrocardiogram, The Affiliated Hospital of Guizhou Medical University, Guiyang, Guizhou Province 550004, China; ^4^Department of Anesthesiology, The Affiliated Hospital of Guizhou Medical University, Guiyang, Guizhou Province 550004, China; ^5^Department of Pathology, The Affiliated Hospital of Guizhou Medical University, Guiyang, Guizhou Province 550004, China; ^6^Department of Cardiovascular Medicine, The Hospital of Qingzhen, Guiyang, Guizhou Province 550004, China; ^7^Department of Respiratory Medicine, The Affiliated Hospital of Guizhou Medical University, Guiyang, Guizhou Province 550004, China; ^8^Department of Cardiovascular Medicine, The Second Affiliated Hospital of Guizhou University of Traditional Chinese Medicine, Guiyang, Guizhou Province 550004, China

## Abstract

**Purpose:**

To investigate the detailed mechanism of 3-iodothyronamine (T1AM) in cell apoptosis and programmed necrosis of hypoxia/reoxygenation- (H/R-) induced H9C2 injury.

**Materials and Methods:**

Cardiomyocyte H9C2 cells were cultured in vitro for the establishment of cardiomyocyte H/R models. Cells were randomly divided into four groups: the control group, H/R group, T1AM pretreatment group, T1AM pretreatment and H/R (6 *μ*m T1AM+H/R) group. The degree of myocardial injury was determined by the detection of the cardiomyocyte inhibition rate by CCK8 and the detection of lactic dehydrogenase (LDH) activity. Cell apoptosis was assessed through TUNEL assay and flow cytometry analysis. The protein level and mRNA level of RIPK1, RIPK3, and CAMKII were detected by western blotting and qRT-PCR.

**Results:**

Compared with the control group, the cell inhibition rate was dramatically elevated in the H/R group. LDH release of cardiomyocytes was significantly increased. Protein and mRNA expressions of RIPK1, RIPK3, and CAMKII were significantly enhanced. Compared with the H/R group, the cell inhibition rate, LDH release, cardiomyocyte necroptosis rate, and protein and mRNA levels of RIPK1, RIPK3, and CAMKII of the T1AM+H/R group were significantly decreased.

**Conclusion:**

Pretreatment with T1AM could alleviate cardiomyocytes' H/R injury and inhibit necroptosis of cardiomyocytes, which might exert a protective function upon activation of the RIPK1/RIPK3 pathway.

## 1. Introduction

Myocardial infarction is one of the most serious diseases, with high mortality and morbidity, throughout the world [1]. The main treatment of myocardial infarction is to restore the blood flow and prevent myocardial injury, but it can lead to myocardial ischemia/reperfusion injury, with arrhythmias, myocardial necrosis, and apoptosis. Oxidative stress, calcium overload, and pH recovery are major mechanisms of myocardial I/R injury [2]. The fate of cardiomyocytes is vital in determining myocardial infarction improvement and hemostasis maintenance. Numerous studies have focused mainly on cardiomyocyte apoptosis. However, accumulating evidence has demonstrated that necroptosis contributes to the incidence and progression of myocardial infarction [3–6]. Nevertheless, the fundamental mechanisms underlying myocardial necroptosis are poorly understood.

RIPK1 is vital for the homeostasis of necroptosis and apoptosis [7]. RIPK1 and RIPK3 have been reported to be critical in the occurrence of necroptosis. RIPK1 has a kinase domain, RHIM, and death domain (DD) protein interaction motifs. RHIM is the main domain that interacts with RIPK3. Preventing necroptosis through RIPK1/RIPK3 inhibition could serve as an effective method to inhibit cell death [8].

3-Iodothyronamine (3-T1AM) is a decarboxylated and deiodinated thyroid hormone (TH) metabolite, which is involved in regulating body temperature, metabolic rate, and cardiac output [9, 10]. T1AM was previously discovered as a meaningful predictor of cardiac morbidity and mortality in patients with chronic heart failure, which may imply that T1AM found in rodents had a direct cardiac effect [11]. Our preliminary studies demonstrated that T1AM could attenuate hypoxia/reoxygenation-induced cardiomyocytes apoptosis via the Akt/FoxO1 pathway [12, 13]. To further the understanding of the role of T1AM in hypoxia-reoxygenation-induced cardiac injury, we extensively analyzed the role of T1AM in attenuating hypoxia-reoxygenation-induced H9C2 cardiomyocyte necroptosis via the RIPK1/RIPK3 pathway.

## 2. Methods and Materials

### 2.1. Cell Cultures

H9C2 cells were purchased from ATCC (Manassas, VA, USA). H9C2 cells were cultured in DMEM (Hyclone, USA) supplemented with 10% fetal bovine serum (FBS, Hangzhou Sijiqing) in a humidified cell incubator providing 5% CO_2_, 95% fresh air at 37°C.

### 2.2. Hypoxia/Reoxygenation Treatment

We chemically synthesized T1AM, purified it with HPLC, and then characterized it using a high-resolution MS approach. The purity of the T1AM was 99.17% based on HPLC analysis. H9C2 cells, a subclone of the original clonal cell line derived from embryonic BD1X rat heart tissue, were randomly divided into four groups: the (a) control group, (b) T1AM pretreatment group, (c) hypoxia/reoxygen (H/R) group, (d) T1AM pretreatment+H/R group. H9C2 cells cultured in a T1AM-free growth medium were used as controls. For the TIAM pretreatment group, 6 *μ*m T1AM was used 0.5 hours before treatment. To establish the H/R injury, H9C2 cells were collected and cultured in the dish at 37°C, 5% CO_2_, and 95% air. Then, the hypoxic H9C2 cells were cultured in DMEM, no glucose (11966025, Gibco) with 95%N_2_, 5%CO_2_, at 37°C for 4 h. Subsequently, the cells were reoxygenated in 5% CO_2_, 95% air for 12 h with DMEM containing 10% FBS. For the T1AM pretreatment+H/R group, 6 *μ*m T1AM was used 0.5 hours prior to H/R treatment. For the TNF-*α*-treated H/R group, 6 *μ*m T1AM and 20 ng/ml TNF-*α* were used 0.5 hours before the H/R treatment. Then, the cells were collected and processed for further analysis.

### 2.3. Cell Viability Assessments

The cell inhibition rate was assessed by CCK8 colorimetry. Briefly, cells were seeded into a 96-well culture plate at a density of 3 × 10^4^/well. H9C2 cells cultured in a T1AM-free growth medium were used as controls. Then, cells in each well were incubated with CCK8 at 37°C for 4 h. The absorbance was determined by a plate reader.

### 2.4. LDH Detection

After treatment, LDH release was detected by using the LDH assay kit. The culture medium was collected and centrifuged at 1000 rpm for 15 min, 40 *μ*l of the supernatant was determined for each group. In a predetermined order, distilled water, pyruvate standard solution, test supernatant, matrix buffer, and coenzyme were added successively, and then, the mixture was incubated at 37°C for 15 min. This was followed by incubation with phenylhydrazine at 37°C for 15 min, followed by mixing with NaOH standard solution for 5 min at room temperature. The absorbance values were measured at 450 nm on the microplate reader.

### 2.5. TUNEL and Flow Cytometry Analysis for Apoptosis

H9C2 cells were seeded into 48-well culture plates at a density of 9 × 10^3^/well at 37°C, 5% CO_2_, and 95% air. After treatment, they were fixed with 4% paraformaldehyde for 0.5 h at room temperature and then infiltrated with 0.2% Triton X-100 for 5 min. After equilibrating with 1x equilibration buffer for 10 minutes, TDT enzyme reaction solution was added followed by reaction for 1 h in the dark. This was followed by washing with PBS 3 times and the addition of Hoechst 33258 dye solution and incubation in the dark for 10 min. After sealing, we observed and photographed the specimens under the fluorescence microscope.

After treatment, the cells were collected, and the number of cells was adjusted to 1 × 10^6^/ml. Cells were incubated with 5 *μ*l Annexin-V-FITC and 5 *μ*l propidium iodide (PI) working solution for 10 min in the dark. Cell fluorescence was measured using a flow cytometer. A control group without fluorescent staining was set up in each group.

### 2.6. Real-Time PCR

Gene expression of RIPK1, RIPK3, and CAMKII in cardiomyocytes was evaluated by reverse transcription qPCR (RT-PCR). Total RNA in cardiomyocytes was extracted with the TRIzol Plus RNA Purification Kit (12183555, Thermo Fisher Scientific, US) according to the manufacturer's instructions. RNA concentration and purity were determined. cDNA was synthesized with a reverse transcription kit (#K1622, Thermo) and amplified with PCR instrument. qRT-PCR was conducted by using a SYBRGreen PCR kit (4309155, Thermo F-415XL) on a StepOnePlus Real-Time PCR System (LightCycle® 480II). The reaction conditions were as follows: preheating at 94°C for 10 min, denaturation at 94°C for 20 s, annealing at 55°C for 20 s, and extension at 72°C for 20 s for 40 cycles. *β*-Actin was used as a control for normalization of RT-qPCR results. Three independent replicates were conducted for this experiment. The following primers were used for RIPK1 qRT-PCR: For, AAAAGGTACAGAGGTGGATT; Rev, GGAGGGTAGAGTATGTGGAA. These primers were used for RIPK3 qRT-PCR: For, CCCGCTCGTGTACTTGGA; Rev, TGGGATGACCCTGTGGAA. And these primers were used for *β*-actin qRT-PCR: For, CACCCGCGAGTACAACCTTC; Rev, CCCATACCCACCATCACACC.

### 2.7. Western Blot

Cardiomyocytes were divided into four groups, lysates of cardiomyocytes were prepared in RIPA buffer, and protein concentration was determined by BCA protein assay (Thermo Fisher Scientific, US). Equal amounts (30 *μ*g) of proteins were separated on 10% SDS-PAGE (100 V for 120 min) and transferred into nitrocellulose membranes (120 min at 100 V) using standard procedures. The binding of nonspecific proteins to the membrane was blocked with blocking buffer (5% nonfat milk) at room temperature for 2 h. The membranes were probed with anti-CaMKII (ab52476, Abcam), anti-RIPK1 (ab106393, Abcam), anti-RIPK3 (sc-374639, Santa Cruz), and anti-*β*-actin (T40104, abmart) at 4°C overnight. After the membrane was washed three times with TBST (20 mM Tris, 500 mM NaCl, and 0.1% Tween 20) for 10 min each time at room temperature, the membrane was incubated with the second antibody (1 : 20000) for 1 h at 37°C. Then, the membrane was washed three times with TBST for 10 min each time at room temperature. Detection was performed using an ECL chemiluminescence kit (PK10003, Proteintech). Finally, the PVDF membrane was placed in the Bio-Rad ChemiDocXRS gel imaging system for photography under exposure, and band intensities were quantified using Image Lab software. Three independent replicates were conducted for this experiment.

### 2.8. Statistical Analysis

Data are shown as means ± SD, unless otherwise stated. SPSS (version 23.0) was used for analysis. One-way ANOVA was used for the overall significance followed by Tamhane's T2 multiple comparison posttest. *P* < 0.05 was considered statistically significant. All data acquisition and analyses were blind.

## 3. Results

### 3.1. T1AM Protected H9C2 Cells from Hypoxia-Reoxygenation-Induced Cell Injury

**Figure 1:**
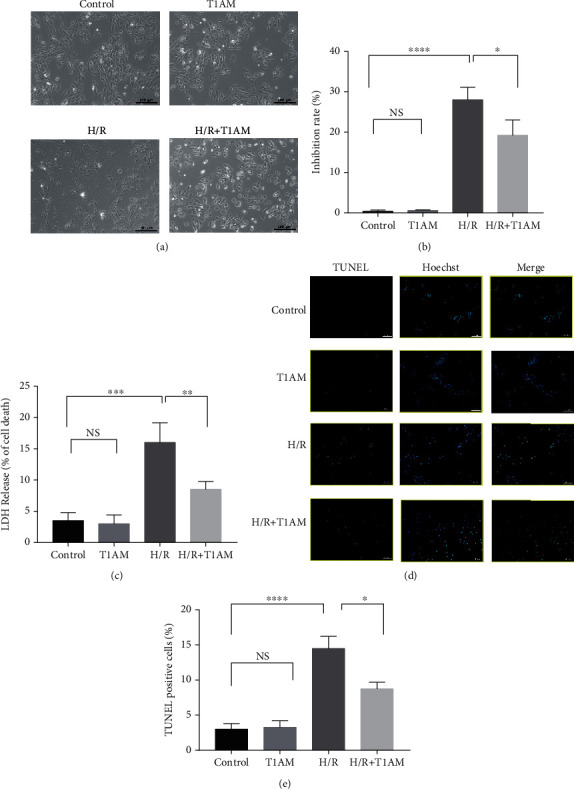
T1AM protects H/R-induced H9C2 cells injury. (a) The effects of T1AM on H/R-induced cell morphology. (b) CCK8 assay (*n* = 4) revealed that T1AM improved H/R-induced H9C2 cell viability. (c) LDH release (*n* = 4) in the culture medium of H9C2 cells exposed to hypoxia 24 h and reoxygenation 4 h treated with 6 *μ*M T1AM. (d) TUNEL stain positivity and Hoechst stain condensed nuclei of H9C2 cells (*n* = 4). (e) Quantification of TUNEL-positive cells. Control: H9C2 cells cultured in normoxia; T1AM: H9C2 cells cultured in normoxia treated with 6 *μ*M T1AM; H/R: H9C2 cells exposed to hypoxia 24 h and reoxygenation 4 h; H/R+T1AM: H9C2 cells exposed to hypoxia 24 h and reoxygenation 4 h treated with 6 *μ*M T1AM.

**Figure 2:**
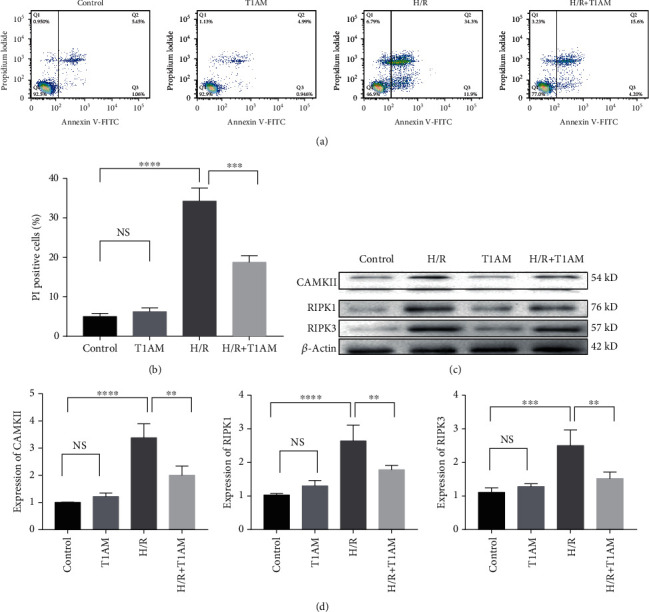
T1AM protects H/R-induced H9C2 cells injury via RIPK1/RIPK3 pathway. (a) Annexin V/PI staining. (b) Quantification of PI-positive cells. (c) Western blot. (d) The band intensities are quantified.

In the control group and in the T1AM pretreatment group, H9C2 cells grew well (Figure 1(a)). However, in the H/R group, H9C2 cells had shrinkage and irregular cell morphology, more drift dead cells and less cell density (Figure 1(a)). Compared with the H/R group, there were fewer dead cells and reduced cell density in the T1AM pretreatment+H/R group (Figure 1(a)). Our data indicated that there was a significantly increased cell inhibition rate after H/R compared with the control group (Figure 1(b)). Compared with the H/R group, the cell inhibition rate decreased in the T1AM pretreatment+H/R group (Figure 1(b)). Our results showed that the LDH leakage was significantly higher in the H/R group compared with the control group (*P* < 0.0001), which could be alleviated by 6 *μ*mol/l T1AM (*P* < 0.05, Figure 1(c)). Our results showed that the cellular apoptosis rate was significantly higher in the H/R group compared with the control group (*P* < 0.05), which could be alleviated by 6 *μ*mol/l T1AM (*P* < 0.05, Figure 1(d)). The results of flow cytometry also confirmed this conclusion (*P* < 0.05, Figures 2(a) and 2(b)).

### 3.2. T1AM Alleviated Hypoxia-Reoxygenation-Induced H9C2 Cell Necroptosis via RIPK1/RIPK3 Pathway

**Figure 3:**
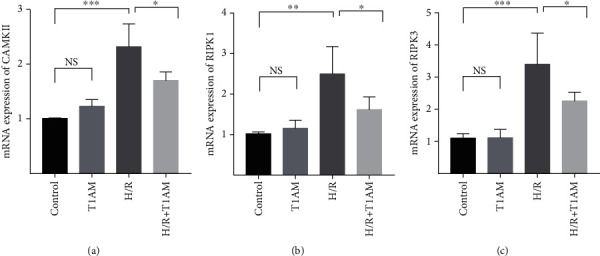
mRNA expression of CAMKII, RIPK1, and RIPK3.

As shown in Figure 2(c), compared with the control group, the protein levels of RIPK1, RIPK3, and CAMKII experienced remarkably increased promotion in the H/R group. However, compared with the H/R group, T1AM (6 *μ*M) significantly reduced RIPK1, RIPK3, and CAMKII protein expressions. As presented in Figure 3, compared with the control group, the mRNA levels of RIPK3 and CAMKII were significantly increased in the H/R group, and the promotion of RIPK1 was also significant (*P* < 0.05). However, the mRNA levels of RIPK1, RIPK3, and CAMKII had a significant reduction in the 6 *μ*M+H/R group. The results of gene expression were similar to those of the protein expression after treatment with 6 *μ*M T1AM in the H/R group.

### 3.3. TNF-*α* Promoted Hypoxia-Reoxygenation-Induced H9C2 Cell Necroptosis Attenuated by T1AM

**Figure 4:**
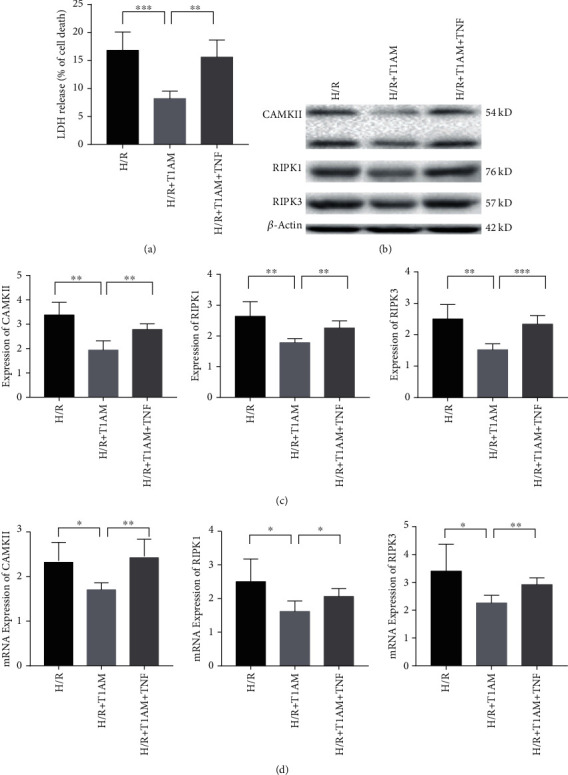
T1AM preconditioning alleviates cardiomyocytes necroptosis by the RIPK1/RIPK3 pathway which can be rescued by TNF-*α*. (a) LDH release. (b) Western blot. (c) The band intensities are quantified. H/R: H9C2 cells exposed to hypoxia 24 h and reoxygenation 4 h; H/R+T1AM: H9C2 cells exposed to hypoxia 24 h and reoxygenation 4 h treated with 6 *μ*M T1AM; H/R+T1AM+TNF-*α*: H9C2 cells exposed to hypoxia 24 h and reoxygenation 4 h treated with 6 *μ*M T1AM and 20 ng/ml TNF-*α*.

T1AM preconditioning alleviated cardiomyocyte necroptosis by the RIPK1/RIPK3 pathway which can be rescued by TNF-*α*. Compared with the H/R+T1AM group, TNF-*α* could induce more H9C2 cells' death (Figure 4(a)). Furthermore, the protein levels of RIPK1, CAMKII, and RIPK3 were detected. Compared with the H/R+T1AM group, the mRNA and protein expressions of CAMKII, RIPK1, and RIPK3 were significantly increased (*P* < 0.05, Figures 4(b)–4(d)).

## 4. Discussion

In this study, we found that T1AM preconditioning plays a protective role in hypoxia-induced cell injury, and this role also improved cell viability, with decreasing LDH release and necroptosis. Multiple compounds were identified as targets of T1AM, including TAAR1, *α*2A adrenergic receptor, and *β*2 adrenergic receptor [14–16]. Scanlan et al. has shown that T1AM administration can lead to bradycardia, which has a negative inotropic effect such as decreased cardiac output in a dose-dependent manner [17, 18]. Our previous study also showed that T1AM has protective effects on hypoxia-reoxygenation induction of AC16 cardiomyocyte apoptosis via inhibition of cardiac metabolism [12]. The negative inotropic properties in the heart make T1AM an optimistic molecule in the prevention of myocardial ischemia-reperfusion injury.

In our study, the expression of RIPK1 and RIPK3 was increased by hypoxia-reoxygenation injury. Furthermore, T1AM preconditioning alleviates cardiomyocyte necroptosis by the RIPK1/RIPK3 pathway which can be rescued by TNF-*α*. A previous work has suggested that necroptosis of cardiomyocytes is a crucial hallmark of myocardial ischemia-reperfusion injury [19]. Necroptosis is a highly regulated cell death that is mainly controlled by RIPK1 and RIPK3 [20]. The activation of necroptosis is mediated by engaging tumor necrosis factor receptor 1 (TNFR1) [21]. Once TNF-*α* binds to TNFR1, prosurvival complex I which included TNFR1-associated death domain (TRADD), TNFR-associated factor 2 (TRAF2), and RIPK1 was recruited [22]. In necroptosis cells, RIPK1 binds to RIPK3 to form necrosome, which phosphorylate and activate RIPK1 and RIPK3, resulting in membrane disruption and cell necroptosis. Hence, the RIPK1 and RIPK3 pathway is important for initiating cell necroptosis in response to TNF-*α* stimulation.

## 5. Conclusion

In conclusion, our data suggest that thyroid hormone metabolite T1AM preconditioning plays a protective role in hypoxia-induced cell injury. Herein, we show that T1AM can alleviate H/R-induced H9C2 cell necroptosis via the RIPK1/RIPK3 pathway. These data might indicate that T1AM is a promising candidate to protect cardiomyocytes from ischemia-reperfusion injury.

## Data Availability

The dataset supporting the conclusions of this article will be available to the Editors and Reviewers upon request.
